# Serum Cytokine Profiles of Melanoma Patients and Their Association with Tumor Progression and Metastasis

**DOI:** 10.1155/2021/6610769

**Published:** 2021-01-28

**Authors:** Xu Wang, Yoel Genaro Montoyo-Pujol, Sandra Bermudez, Gonzalo Corpas, Aurelio Martin, Francisco Almazan, Teresa Cabrera, Miguel Angel López-Nevot

**Affiliations:** ^1^Servicio de Análisis Clínicos e Inmunología, Hospital Universitario Virgen de Las Nieves, Avda. Fuerzas Armadas s/n, Granada 18014, Spain; ^2^Programa de Doctorado en Biomedicina, Universidad de Granada, Granada, Spain; ^3^Servicio de Anatomía Patológica, Hospital Universitario Virgen de Las Nieves, Avda. Fuerzas Armadas s/n, Granada 18014, Spain; ^4^Servicio de Dermatología, Hospital Universitario Clinico San Cecilio (PTS), Avenida de la Investigación s/n, Granada 18014, Spain; ^5^Departamento Bioquímica, Biología Molecular e Inmunología III, Universidad de Granada, Granada, Spain; ^6^Instituto de Investigación Biosanitaria I Ibs. GRANADA, Avda de Madrid 15, Pabellón de Consultas Externas 2, Granada 18012, Spain

## Abstract

**Purpose:**

Previous studies have shown that melanoma cells produce excessive levels of cytokines, which have various biological roles during melanoma development. The aim of this study was to expand the profile of serum cytokines, chemokines, growth factors, and angiogenic factors that are associated with melanoma, to find more cytokines with abnormal concentrations in melanoma patients, to identify whether the level of cytokines correlated with prognostic variants, such as Breslow thickness and BRAF mutation, and, finally, to find out the cytokines that play important roles during melanoma development.

**Materials and Methods:**

Multiplex immunobead assay technology and 45-plex immunoassays ProcartaPlex™ kits were used to simultaneously compare the levels of cytokines, growth factors, angiogenic factors, and chemokines between the serum of healthy patients (*n* = 30) and those with melanoma (*n* = 72). Data were analyzed according to the clinical characteristics of the designated patient subgroups.

**Results:**

Compared to the control group, melanoma patients had higher levels of VEGF-A, PDGF-BB, IL-1RA, PIGF-1, IFN-*γ*, TNF-*α*, MIP-1*α*, and SCF, but lower levels of BDNF, SDF-1*α*, MCP-1, Eotaxin, EGF, and IL-7. Furthermore, the levels of TNF-*α* (*P*=0.320, *r* = 0.019), IFN-*γ* (*P*=0.311, *r* = 0.023), VEGF-A (*P*=0.014, *r* = 0.337), and BDNF (0.004, *r* = −0.391) showed a significant correlation with Breslow thickness. IL-7 was of lower levels in patients harboring BRAF mutants. Melanoma patients with high levels of MIP-1*α* and MCP-1 showed the poorest overall survival.

**Conclusions:**

We found that the levels of VEGF-A and PDGF-BB in the serum of both primary and metastatic melanoma patients are elevated. TNF-*α*, IFN-*γ*, and VEGF-A presented a positive correlation with Breslow thickness, whereas BDNF showed a negative association. MIP-1*α* and MCP-1 correlated negatively with survival. In addition, lower levels of IL-7 were found in patients harboring BRAF mutants. These findings indicate that these cytokines may play critical roles in the progression of melanoma.

## 1. Introduction

Melanoma is a potentially lethal malignancy that occurs when melanocytes undergo DNA damage in response to ultraviolet light (UVB, UVC) [1]. In the United States, there are an estimated 21.8 melanoma cases per 100,000 inhabitants, with 76,380 reported in 2016 alone, representing 4.5% of all malignant tumors and 10,130 cases of melanoma-related deaths (https://seer.cancer.gov/). The prognosis of melanoma is variable. When melanoma is discovered at an early stage, including primary melanoma with a Breslow index <1 mm (Stage IA), surgical treatment leads to a favorable prognosis. However, when the same primary melanoma shows regional lymph node metastasis and presence of satellitosis (Stage III), the prognosis is poor [2–5]. Melanomas are also prone to chemotherapy and radiotherapy resistance, leading to a poor prognosis. Therefore, finding more immunotherapy targets is very important for the treatment of melanoma.

Studies have highlighted that the alteration of the cytokine levels in melanoma patients can affect the disease progression, response to the therapeutic, and the outcome of the disease [6, 7]. For example, high concentrations of fibroblast growth factor (bFGF), interleukin- (IL-) 8, transforming growth factor (TGF-*β*), granulocyte colony-stimulating factor (GCSF), and platelet-derived growth factor (PDGF) are detected in melanoma patients. These factors stimulate angiogenesis in stromal or infiltrating host cells, leading to the development of neovasculature that facilitates the transportation of nutrients that are required for melanoma growth [8–10]. In addition, high concentrations of IL-10 are observed in patients with Stage II and IV melanomas and contribute to the downmodulation of antitumor responses [11], while abnormally high concentrations of IL-6 are associated with a poor response and outcome and contribute to the development and progression of early-stage melanoma [12–14]. The expression level of vascular endothelial growth factor (VEGF) presents a negative association with outcomes in patients with mucosal melanoma [15]. So it is important to find out the critical cytokines that contribute to tumor progression for a better understanding of the process of tumor invasion and metastasis to get better results of antitumor chemotherapy and immunotherapy.

However, these studies were limited to only explore a single family of cytokines, while the tumor progression is often influenced by a variety of cytokines. Clinically, usage of the single cytokine inhibitor that plays an important role in the development of melanoma shows limited effectiveness, and the treatment strategy of multitarget inhibitor shows more therapeutic benefits. Therefore, it is very important to find out more cytokines that can be used as multitarget therapeutic targets [16]. In this study, we used Luminex xMAP technology and 45-plex immunoassay ProcartaPlex™ kits to expand the profile of serum cytokines, in order to explore more cytokines with abnormal concentrations in melanoma patients, evaluate correlations between the cytokine levels and Breslow thickness and BRAF mutation, identify whether the concentrations of cytokines correlated with the survival rate, and determine the critical cytokines in the progression of melanoma.

## 2. Materials and Methods

### 2.1. Patients/Controls

Serum samples were obtained from 72 patients diagnosed with Stage I–IV melanomas. Tumor stages were defined from biopsy samples according to the criteria of the American Joint Committee on Cancer TNM staging system (AJCC 2018), through the Dermatology Service of Virgen de las Nieves University Hospital of Granada, Spain. Exclusion criteria were as follows: cardiac disease, autoimmune disease, and diabetes mellitus. The control subjects included 30 age- and gender-matched healthy donors that were obtained from the blood bank of Granada. All healthy donors had no indications of immune-related diseases. Samples were collected from all subjects from 2013 to 2015. Patient samples were obtained prior to primary metastasis melanoma excision. All subjects provided written informed consent prior to sample collection. The study protocol was approved by the hospital institutional review board and ethical committee. The clinicopathological characteristics of the patients are summarized in Table 1.

### 2.2. Serum Collection and Storage

Peripheral blood (20 ml) samples were obtained from patients and healthy donors using standardized phlebotomy procedures. Operating procedures and standards were maintained throughout the sample collection. Blood samples were collected in the absence of anticoagulants and allowed to coagulate for 20 to 30 min at room temperature. Samples were separated by centrifugation, aliquoted, and stored at −80°C prior to analysis.

### 2.3. Cytokine, Chemokine, Angiogenic Factors, and Growth Factor Measurements

The concentrations of 45 soluble factors were measured in the serum samples of all subjects using commercially available human Cytokine/Chemokine/Growth Factor 45-plex immunoassay ProcartaPlex™ kits (eBioscience, Vienna, Austria). Measurements were performed on a Luminex platform (Life Technologies, Frederick, MD), using a Luminex^TM^ 200 technology instrument (Luminex^TM^ Copr., Austin, TX, USA) according to the manufacturer's instructions. The soluble factors assayed included the following: (1) inflammasome activation: IL-1-*β*, IL-1-*α*, IL-1RA, and IL-18; (2) TH1 cytokines: IL-2, IFN-*γ*, TNF-*α*, and TNF-*β*; (3) interferons: INF-*α*; (4) IL-2 family: IL-7, IL-9, IL-12p70, IL-15, and IL-27; (5) TH2 cytokines: IL-4, IL-5, IL-10, IL-13, and IL-31; (6) IL-6 family: IL-6 and LIF; (7) TH17 cytokines: IL-17A, IL-21, IL-22, and IL-23; (8) chemokines: 8.1.- CCL : MCP-1 (CCL2), MIP-1*α* (CCL3), MIP-1*β* (CCL4), RANTES (CCL5), Eotaxin (CCL11); 8.2.- CXCL : GRO-*α* (CXCL1), IL8 (CXCL8), IP-10 (CXCL10), and SDF1*α* (CXCL12a); (9) growth factors: SCF, GM-GSF, EGF, HGF, FGF-2, NGF*β*, and BDNF; (10) angiogenic factors: VEGF-A, VEGF-D, PDGF-BB, and PlGF-1. Calibrations and validations were performed prior to measurements and repeated on a monthly basis. Mean fluorescence intensities were calculated from duplicate samples to determine the limit of detection. Values below this limit were designated as 50% of the lowest limit.

### 2.4. Statistical Analysis

The Shapiro–Wilk test was used to analyze the normality of distributions. When the data did not exhibit a normal distribution, nonparametric statistical analyses were performed. The Mann–Whitney test was used to compare differences in the median cytokine levels between patients with BRAF mutants and BRAF wild-type (WT) patients. The Kruskal–Wallis test and Dunn's multiple comparison test were used to determine differences between healthy donors, primary melanoma, and metastatic melanoma. The Spearman's rank correlation coefficient was used to assess the correlation between the Breslow thickness of 53 patients with primary tumors and the concentration of serum cytokines. The relationship between cytokine concentrations and the survival rates of each cytokine were stratified into two groups based on the median concentration. The follow-up time was defined from the date of blood removal of the analyzed sample to the date of final follow-up or death through the DIRAYA medical records of the hospital. Kaplan–Meier curves were used for survival analysis. *P*-values <0.05 were considered significant. Data were analyzed using commercially available statistical software programs (SPSS 22, SPSS, Inc., Chicago, IL; GraphPad Prism 6.02, GraphPad Software, La Jolla. CA).

## 3. Results

### 3.1. Cytokine Concentrations in Melanoma Patients

Serum samples were obtained from 72 patients with melanoma. The distribution of clinical stages was as follows: Stage I (*n* = 42); Stage II (*n* = 11); Stage III (*n* = 11); Stage IV (*n* = 8). To investigate the involvement of specific cytokines and chemokines during melanoma development, the serum levels of each soluble factor were compared across different stage melanoma patients (Stages I and II, *n* = 53) and metastasis (Stages III and IV, *n* = 19) melanoma groups and compared to healthy controls (*n* = 30).

**Figure 1:**
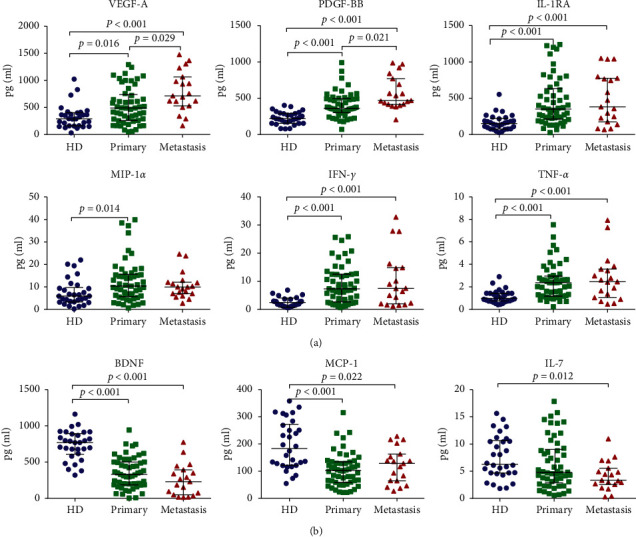
Analysis of cytokine levels in the serum samples of melanoma patients and healthy controls. Serum was collected from 53 primary melanoma patients and 19 metastatic melanoma patients and compared to the sera of 30 healthy donors. Measurements were performed using the 45-plex immunoassay system. (a) Cytokines showing higher concentrations in melanoma patients than those of healthy donors. (b) Cytokines showing lower concentrations in melanoma patients than those of healthy donors. Horizontal lines indicate the median of each group.

From these analyses, higher concentrations of VEGF-A, PDGF-BB, IL-1RA, PIGF-1, IFN-*γ*, TNF-*α*, MIP-1*α*, and SCF (Figure 1, Table 2) were observed in melanoma patients. In contrast, lower concentrations of BDNF, SDF-1*α*, MCP-1, Eotaxin, EGF, and IL-7 were observed in the serum of melanoma patients compared to healthy controls (Figure 1, Table 3). Of the elevated cytokines, VEGF-A and PDGF-BB showed significant differences between the three groups, with higher concentrations in metastatic melanoma (Stages III and IV) compared to primary melanoma (Stage I and Stage II). In addition, IL-1RA, PIGF-1, IFN-*γ*, and TNF-*α* levels were significantly elevated in primary melanoma and metastatic melanoma patients compared to healthy donors but showed no differences between primary melanoma and metastatic melanoma. MIP-1*α* and SCF levels were significantly higher in primary melanoma patients than those in healthy donors. Decreased concentrations of BDNF, SDF-1*α*, MCP-1, Eotaxin, and EGF were observed in primary melanoma and metastatic melanoma patients compared to healthy subjects. However, the levels of these soluble factors did not differ between primary melanoma and metastasis melanoma patients. IL-7 levels were significantly lower in metastatic melanoma patients than those in healthy controls.

No significant differences were observed in the levels of LIF, IP-10, HGF, MIP-1*β*, IL-18, RANTES, IL-1*β*, VEGF-D, GRO-*α*, IL-4, IL-5, IL-2, IL-12p70, IL-13, bNGF, IL-21, and FGF-2. The levels of IL-6, IL-8, IL-10, IL-17A, IL-27, IL-31, GM-CSF, IFN-*α*, IL-9, VEGF-D, TNF-*β*, NGF*β*, IL-23, IL-22, and IL-1*α* were below the threshold of detection in both melanoma patients and healthy controls.

### 3.2. Cytokine Concentrations and Breslow Thickness

**Figure 2:**
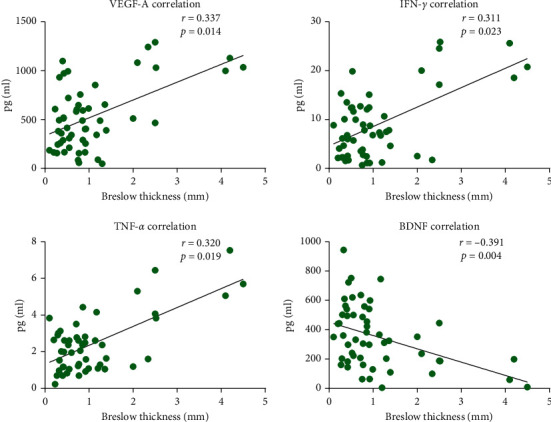
Cytokine concentrations and Breslow thickness. Correlation between the concentration (pg/ml) of soluble factors in primary melanoma patients (*n* = 53) and Breslow thickness (mm). Spearman's correlation coefficients (r) show the strength of correlation. *P* < 0.05 was considered significant.

The Breslow thickness is the strongest predictor of patient survival and can be used to define the depth of tumor invasion from the granular stratum to the deepest penetrating melanoma cells. Using the AJCC melanoma staging system for primary melanoma, tumors ≤1.0 mm are designated as low-risk with 10-year survival rates of 92%; tumors of 1.01 to 2.0 mm have 10-year survival rates of 80%; tumors measuring 2.01–4.0 mm have 10-year survival rates of 63%; tumors ≥4 mm have 10-year survival rates of 50% [4,17]. We identified 3 cytokines that are positively correlated with increased thickness: TNF-*α* (*P*=0.320, *r* = 0.019), IFN-*γ* (*P*=0.311, *r* = 0.023), and VEGF-A (*P*=0.014, *r* = 0.337). In contrast. BDNF (0.004, *r* = −0.391) negatively correlated with Breslow thickness (Figure 2).

### 3.3. BRAF and Cytokine Levels

**Figure 3:**
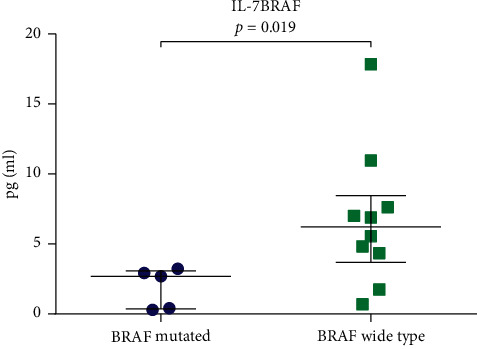
Cytokine analysis in the serum samples of melanoma patients with BRAF mutations (*n* = 5) *vs.* BRAF WT (*n* = 10). Differences in the levels of IL-7. Horizontal lines indicate the median of each group.

We next investigated the relationship between cytokine levels and the development of metastatic melanoma (Stages III and IV). BRAF is the most frequent mutation observed in melanoma patients, with BRAF testing recommended for advanced melanoma patients to permit the selection of the optimal treatment regimen [18]. The study cohort consisted of 19 advanced melanoma patients (11 with Stage III and 8 with Stage IV), among which 15 underwent BRAF testing, with 5 patients harboring BRAF-mutants and 10 patients showing a WT BRAF mutant status. The concentrations of cytokines were compared between BRAF mutant and BRAF WT patients using a Mann–Whitney test, as the data showed a nonnormal distribution. From these analyses, only IL-7 (*P*=0.019, Figure 3, Table 4) was of significantly lower levels in BRAF mutants vs. WT BRAF patients.

### 3.4. Predictive Values of Cytokine Levels following Survival Analysis

**Figure 4:**
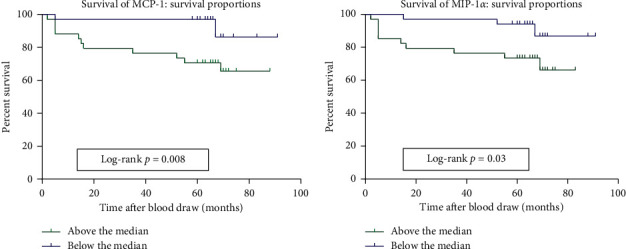
Survival curves for melanoma patients based on the median levels of each cytokine.

As Breslow thickness represents the strongest prognostic factor of survival for primary melanoma, we next sought to investigate if the concentration of cytokines in the serum samples of melanoma patients correlated with patient survival. The 5-year age-standardized relative survival of melanoma patients has significant differences between different regions. In Europe, the highest relative survival rate is in Northern Ireland (90.7%), the lowest is in Bulgaria (49.6%). In addition, it is different among different stages of melanoma. In the United States, the 5-year relative survival for primary melanoma without lymph node involvement is 98% in melanoma patients with Stage I and 90% in Stage II [19]. In our study, melanoma patients were stratified into two groups based on the median cytokine concentrations, namely, those with high cytokine levels above the median and those below the median. Kaplan–Meier curves were plotted for the statistical analysis of patient survival. MIP-1*α* (*P*=0.03) and MCP-1 (*P*=0.008) were shown to influence survival, with melanoma patients with the poorest survival rates showing high concentrations of MIP-1*α* and MCP-1 (Figure 4). No positive correlation with patient survival was observed.

## 4. Discussion

Cytokines are known to regulate the developmental processes of tumors [6, 8, 11]. In this study, melanoma patients showed higher levels of VEGF-A, PDGF-BB, IL-1RA, PIGF-1, IFN-*γ*, TNF-*α*, MIP-1*α*, and SCF, but decreased levels of BDNF, SDF-1*α*, MCP-1, Eotaxin, EGF, and IL-7 than those in healthy patients. Among them, VEGF-A and PDFG-BB were higher in advanced (Stages III and IV) melanoma patients than those in early (Stages I and II) patients and healthy donors and were positively correlated with Breslow thickness. VEGF-A signaling is known to stimulate cell proliferation and neovasculature formation that facilitates tumor growth, disease progression, and metastatic processes [20–22]. VEGF/VEGFR inhibitors, including bevacizumab, have been approved as cancer therapeutics and can improve patient prognosis in specific cancers. Their use has, however, been limited by their low rates of therapeutic efficacy [20]. PDGF-BB is a non-VEGF angiogenic factor that can stimulate cell proliferation and the formation of capillaries to promote tumor metastasis [23]. In this study, higher levels of PDGF-BB were evident in metastatic melanoma compared to primary melanoma patients. This may explain in part why the use of VEGF inhibitors as monotherapies shows only limited effectiveness, as more than a single angiogenic factor may mediate tumor monotherapy. More recent antiangiogenic treatment regimens should therefore focus on the use of multitargeted inhibitors to simultaneously block several angiogenic pathways. In this regard, the increased benefits of targeting both VEGFR and PDGFR pathways has been reported in B16/PDGF-BB tumors [16, 23].

Breslow thickness is an important predictor of patient survival and is an important parameter of AJCC TNM classification for primary melanoma patients. A higher Breslow thickness indicates poorer mortality and a more advanced tumor stage [4]. We, therefore, explored the relationship between cytokine levels and Breslow thickness. We observed higher levels of IFN-*γ*, TNF-*β*, and VEGF-A and lower levels of BDNF that correlated with increased Breslow thickness. The negative correlation of BDNF highlights its protective role during melanoma development. BDNF is a key mediator of the hypothalamus-adipocyte axis, with studies indicating that this neuroendocrine axis shows antiobesity and anticancer functions in animal models of melanoma and colon cancer. The recombinant adeno-associated viral vector-mediated overexpression of BDNF was also used to reduce angiogenesis and decrease the proliferation of mouse breast cancer cells to prevent their metastasis. BDNF has since been shown to hold therapeutic significance for a range of cancers [24, 25]. IFN-*γ* and TNF-*β* were also elevated in melanoma patients, with a positive correlation observed between their concentrations and tumor progression, as observed in cases of uveal melanoma [6, 7, 26].

Upon comparison of the levels of cytokines between BRAF mutant and BRAF wild-type patients, only IL-7 showed lower levels in patients with BRAF mutations. Of interest, the concentration of IL-7 decreased with tumor stage, although significant differences in its expression were limited to metastasis and healthy donor groups. IL-7 is a cytokine from the IL-2 family, with IL-2 treatment in low or high doses in patients with metastatic melanoma identified as successful immunotherapy. Therefore, decreasing the levels of IL-7 may be beneficial to prevent tumor development and metastasis. While IL7 is an important candidate immunomodulator for cancer therapy, its use in the clinic is limited by its short half-life in the serum. Accordingly, direct treatments have been surpassed by recombinant cytokines. In this regard, Feng et al. 2017 used recombinant human IL-7/HGF*β* hybrid cytokines to enhance antitumor immunity in mice. Yinhon et al. 2016 used recombinant IL-7/IL-15 hybrid cytokines for the treatment of melanoma in mice, with both showing promising clinical efficacy [27–29].

In this study, several cytokines related to melanoma progression were identified. These cytokines were divided into high or low groups based on their median values to explore their prognostic value. We found that high levels of MCP-1 and MIP-1*α* were related to poor survival rates. Monocyte chemotactic protein 1 (MCP-1, also termed CCL2) is produced by monocytes, fibroblasts, epithelial cells, and several tumor cells and plays an important role in the recruitment and activation of macrophages and monocytes during antitumor immunity [30]. MCP-1 plays a dual role in cancer development, with studies indicating that, in melanoma, high levels of MCP-1 prevent tumor growth, but low or intermediate levels favor tumor progression [31]. It is interesting to note that although low levels of MCP-I contribute to tumor progression, high concentrations of MCP-1 correlate with poorer overall survival, consistent with our analysis. This may be related to the fact that MCP-1 can upregulate prosurvival signaling pathways [32]. Macrophage inflammatory protein-1 alpha (MIP-1*α*) is a chemokine from the RANTES family that negatively correlates with survival in patients with multiple myeloma [33]. In this study, MIP-1*α* correlated with the survival rates of melanoma patients. In studies of carotid aneurysms, MCP-1 was found to play an important role in promoting inflammatory intra-aneurysmal tissue healing, which could be blocked with MIP-1*α* and MIP-2 antibodies [34]. Our results suggest that MIP-1*α* and MCP-1 hold predictive value in melanoma, with their role as potential therapeutic agents now warranting further investigation.

Some limitations of this study should be noted. First, the number of experimental samples was low, limiting the analysis. Secondly, the distribution of samples was unequal across all tumor stages. The MNT stage system classifies primary melanoma into four stages, with the majority of samples at Stage I in this study. This may have limited the discovery of cytokines that correlate with Breslow thickness. However, this is a common limitation, as melanoma is a skin disease that is easily discoverable by patients. As such, patient samples that can be collected at late tumor stages are sparse, often resulting in an uneven grouping for analyses.

## 5. Conclusions

We found several cytokines with abnormal concentrations in melanoma patients. Specially, the levels of VEGF-A and PDGF-BB in the serum of both primary and metastatic melanoma patients are elevated; it is suggested that these two cytokines play important roles in the development, invasion, and metastasis of melanoma. In addition, TNF-*α*, IFN-*γ*, and VEGF-A correlated positively with Breslow thickness, while BDNF showed a negative association. MIP-1*α* and MCP-1 demonstrated a negative correlation with survival. Lower levels of IL-7 were found in patients harboring BRAF mutants. Those factors not only may correlate with the tumor progression but also affect the outcome of patients with melanoma. Further study of the mechanism of the increase in these cytokines may lead to greater insight into the development and invasion of melanoma and find a better treatment strategy to inhibit tumor growth.

## Figures and Tables

**Table 1:** Patients and clinical characteristics.

Baseline characteristics	Melanoma patients	Healthy donors
Subjects	72	30
Age, median (range)	55 (24–88)	53 (27–85)

Sex		
Male	35 (48.6%)	15 (50%)
Female	37 (51.4%)	15 (50%)

Melanoma stage, *n* (%)		
Stage I	42(58.33%)	
Stage II	11(15.28%)	
Stage III	11 (15.28%)	
Stage IV	8 (11.11%)	

Breslow thickness		
Thickness ≤1 mm	37 (69.8%)	
1.01 mm–2 mm	8 (15.1%)	
2.01 mm–4 mm	5 (9.7%)	
>4.01 mm	3 (5.4%)	

5-year survival		
Alive, *n* (%)	55 (76.4%)	
Deceased, *n* (%)	17 (23.6%)	

**Table 2:** Elevated levels of soluble factors in melanoma patients vs. healthy controls.

Cytokine	Median (range), pg/ml	Significance
VEGF-A		
Healthy donors	290.44 (31.1∼1022.19)	*P* ^3^=0.029
Primary melanoma	487.93 (48.51∼1290.95)	*P* ^1^=0.016
Metastasis melanoma	713.74 (162.5∼1474.77)	*P* ^2^=0.001

PDGF-BB		
Healthy donors	218.10 (77.95∼401.93)	*P* ^3^=0.021
Primary melanoma	360.45 (71.02∼990.06)	*P* ^1^=0.001
Metastasis melanoma	468.29 (204.61∼986.70)	*P* ^2^=0.001

IL-1RA		
Healthy donors	151.24 (26.39∼552.77)	
Primary melanoma	350.753 (26.77∼1240.28)	*P* ^1^ < 0.001
Metastasis melanoma	381.37 (67.86∼1049.55)	*P* ^2^ < 0.001

PIGF-1		
Healthy donors	4.56 (0.06∼16.02)	
Primary melanoma	17.64 (1.94∼76.96)	*P* ^1^ < 0.001
Metastasis melanoma	13.48 (0.91∼38.53)	*P* ^2^ < 0.001

IFN-*γ*		
Healthy donors	1.80 (0.61∼6.86)	
Primary melanoma	7.32 (0.65∼25.86)	*P* ^1^ < 0.001
Metastasis melanoma	7.43 (1.13∼32.85)	*P* ^2^ < 0.001

TNF-*α*		
Healthy donors	0.97 (0.41∼2.9)	
Primary melanoma	2.37 (0.23∼7.53)	*P* ^1^ < 0.001
Metastasis melanoma	2.45 (0.46∼7.93)	*P* ^2^ < 0.001

MIP-1*α*		
Healthy donors	5.877 (0.19∼20.11)	
Primary melanoma	10.37 (0.59∼39.91)	*P* ^1^=0.014
Metastasis melanoma	9.90 (2.72∼24.59)	

SCF		
Healthy donors	4.00 (0.67∼8.23)	
Primary melanoma	6.95 (0.77∼43.51)	*P* ^1^=0.007
Metastasis melanoma	4.70 (0.68∼12.59)	

Serum samples were screened using 45-plex multiplexed cytokine assays. Serum biomarkers showing differences between melanoma patients and healthy control groups are presented as the median (range) (pg/ml). *P*^1^, significant differences between healthy donors and primary melanoma patients. *P*^2^, significant differences between healthy donors and metastasis melanoma patients. *P*^3^, significant differences between primary melanoma and metastatic melanoma patients.

**Table 3:** Soluble factors showing decreased levels in melanoma patients vs. healthy controls.

Cytokine	Median (range), pg/ml	Significance
BDNF		
Healthy donors	769.86 (320.02∼1161.64)	
Primary melanoma	332.13 (4.70∼943.70)	*P* ^1^ < 0.001
Metastatic melanoma	230.67 (14.62∼774.21)	*P* ^2^ < 0.001

SDF-1*α*		
Healthy donors	600.59 (398.09∼857.74)	
Primary melanoma	384.47 (197.16∼825.62)	*P* ^1^ < 0.001
Metastatic melanoma	423.54 (205.60∼601.79)	*P* ^2^ < 0.001

MCP-1		
Healthy donors	183.05 (54.91∼358.44)	
Primary melanoma	102.12 (21.67∼315.81)	*P* ^1^ < 0.001
Metastatic melanoma	128.53 (26.65∼228.36)	*P* ^2^=0.022

Eotaxin		
Healthy donors	76.733 (13.76∼144.17)	
Primary melanoma	43.98 (5.8∼114.72)	*P* ^1^=0.008
Metastatic melanoma	39.98 (5.62∼124.05)	*P* ^2^=0.003

EGF		
Healthy donor	72.34 (2.91∼123.27)	
Primary melanoma	26.86 (3.49∼136.14)	*P* ^1^=0.011
Metastasis melanoma	16.24 (2∼89.45)	*P* ^2^=0.001

IL-7		
Healthy donor	6.29 (1.83∼15.64)	
Primary melanoma	4.80 (0.58∼17.84)	
Metastasis melanoma	3.35 (0.29∼10.95)	*P* ^2^=0.012

**Table 4:** Cytokine levels and BRAF status.

Cytokine	Median (range), pg/ml	Significance
IL-7		
BRAF mutant	2.68 (0.29∼3.22)	*P*=0.019
BRAF wild-type	6.22 (0.68∼17.85)	

## Data Availability

The data used in the study are available on request to the corresponding author.

## Abbreviations

BDNF: Brain-derived neurotrophic factor
FGF-2: Fibroblast growth factor-2
EGF: Epidermal growth factor
GM-GSF: Granulocyte-macrophage colony-stimulating factor
GRO-*α* (CXCL1): Growth-regulated oncogene-*α*
HGF: Hepatocyte growth factor
IL: Interleukin
INF-*α*: Interferon-gamma
IP-10 (CXCL10): Interferon gamma-induced protein 10
LIF: Leukemia Inhibitory Factor
MCP-1 (CCL2): Monocyte chemoattractant protein
MIP-1*α* (CCL3): Macrophage inflammatory protein-1 alpha
NGF*β*: Nerve growth factor-beta
PDGF: Platelet-derived growth factor
PlGF-1: Placental growth factor
SCF: Stem cell factor
SDF1*α* (CXCL12a): Stromal cell-derived factor-1*α*
TNF-*β*: Tumor necrosis factor
VEGF: Vascular endothelial growth factor.
